# Postpartum septic shock presenting as symmetrical peripheral gangrene: A rare entity

**DOI:** 10.12669/pjms.39.3.7277

**Published:** 2023

**Authors:** Wajeeha Batool, Shabnam Naveed, Sulhera Khan, Syed Masroor Ahmed

**Affiliations:** 1 Wajeeha Batool, FCPS. Resident, Department of Medicine, Jinnah Postgraduate Medical Centre Karachi, Pakistan; 2 Shabnam Naveed, MCPS, FCPS. Associate Professor, Department of Medicine, Jinnah Postgraduate Medical Centre Karachi, Pakistan; 3 Sulhera Khan, FCPS. Resident, Department of Medicine, Jinnah Postgraduate Medical Centre Karachi, Pakistan; 4 Syed Masroor Ahmed, FCPS. Professor, Department of Medicine, Jinnah Postgraduate Medical Centre Karachi, Pakistan

**Keywords:** Symmetric peripheral gangrene, Disseminated Intravascular Coagulation postpartum sepsis, gangrene

## Abstract

Symmetric peripheral gangrene (SPG) is a rare clinical entity defined as ischemia of peripheral parts of the body without underlying vaso-occlusive disease. Its pathogenesis is unknown, but it is seen from previous reports that SPG is a sequel of underlying Disseminated Intravascular Coagulation (DIC). We report a case of a middle-aged woman who developed high-grade fever followed by painful black discoloration of the digits of four limbs, few days after spontaneous vaginal delivery at home. The patient developed septic shock. However, peripheral pulses were palpable and radiologic and laboratory investigations did not show any evidence of vessel occlusion. The patient had neutrophilic leukocytosis and a deranged clotting profile. Blood culture revealed growth of Staphylococcus Aureus and Pseudomonas Aeruginosa. The patient was diagnosed with SPG due to postpartum sepsis and DIC. She was managed with fluids, antibiotics, aspirin, and heparin but unfortunately, the patient underwent amputation of limbs due to irreversible ischemia. Therefore, prompt diagnosis and management of SPG are crucial to prevent mortality and morbidity.

## INTRODUCTION

Symmetric peripheral gangrene (SPG) is a rare debilitating clinical phenomenon defined as symmetrical ischemia and gangrene in peripheral parts of the body in the absence of occlusive vascular disease.^1^ It has a multifactorial etiology. The exact pathogenesis of SPG is unknown, but the reported cases suggest that it is the most characteristic sequelae of sepsis and disseminated intravascular coagulation (DIC).^2^,^3^ The initial presentation of the disease is the appearance of pallor, cyanosis, and pain in extremities followed by dry gangrene and amputation of affected body parts if treatment is delayed.^2^ SPG carries a mortality of 40% and in survivors, the rate of limb amputation is 70%.^3^ Occasionally, SPG is reported during pregnancy and the postpartum period. The commonly reported precipitating factors are sepsis and DIC.^3^ We present a case of symmetrical postpartum SPG involving all four limbs as a consequence of DIC following sepsis.

## CASE PRESENTATION

A middle-aged female presented via the emergency department with complaints of high-grade fever and black discoloration of her hands, feet, and tip of the nose for seven days. She had spontaneous vaginal delivery at home by an unskilled midwife ten days prior to presentation. The patient was well two days postpartum after which she developed high-grade fever, followed by bluish discoloration of finger and toe tips, associated with tingling, paresthesia, and burning sensation. The discoloration progressed to involve the forearms, ankles, and the tip of the nose. The patient also developed severe pain in the affected areas temporarily subsided with analgesics. The patient denied claudication, Raynaud phenomenon, arthralgia, or rash. Her past history, family, travel and drug history were insignificant.

**Fig.1:**
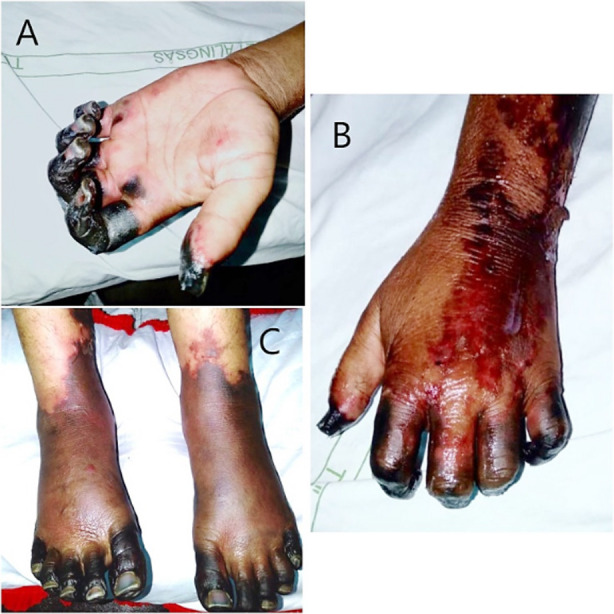
Hands and feet of the patient with digital gangrene and nail discoloration. (A) Gangrene of fingers and thumb with clear line of demarcation. (B) Pre-gangrenous changes involving the dorsum of the hand. A large erythematous, necrotic patch with desquamation of skin. (C) Acro-cyanosis of toes and purpuric erythema with a dusky hue extending from the dorsum of feet up to lower one-third of legs.

On physical examination, she was alert and well-oriented with a blood pressure of 90/60 mmHg, a regular pulse of 112 beats/minute, a respiratory rate of 20 breaths/minute, and a temperature of 102°F. She was immediately identified with systemic inflammatory response syndrome (SIRS). She had black discoloration on the tip of her nose, associated with loss of sensation. Her hands were cold and painful, and her fingers were swollen with dry gangrene extending up to the metacarpophalangeal joints of all fingers and thumbs bilaterally associated with a restricted range of motion (Fig.1A).

The dorsum of the hand showed a necrotic erythematous patch with superficial skin sloughing. (Fig.1B). Similar changes were seen in feet with acro-cyanosis extending up to the lower one-third of the legs. (Fig.1C). The nailbeds had blue-black discoloration. Peripheral pulses were palpable with full volume in her upper and lower limbs without radio-radial or radio-femoral delay. Per vaginal examination did not reveal any discharge or blood clots.

Investigations demonstrated microcytic hypochromic anemia, thrombocytopenia, neutrophilic leukocytosis, raised serum lactate and inflammatory markers, deranged coagulation profile, elevated D-dimers and low fibrinogen levels. All the investigations are summarized in Table-I. Diagnostic screening of sepsis was done including blood and urine cultures. Doppler imaging of all four limbs showed normal flow in arteries, with no evidence of venous thrombi. Transvaginal ultrasound ruled out any retained products of conception. The patient had a normal chest X-ray, echocardiography, and a negative autoimmune profile.

**Table-I:** Laboratory results for blood tests.

Complete blood count	On Admission	5^th^-day post-admission
Haemoglobin(g/dl)	8.5	8.7
Mean corpuscular volume(fl)	62.7	64.8
Hematocrit %	25.5	26.1
Platelets (10^9^/L)	107	156
White blood cells(10^9^/L)	34	16
Neutrophil %	88	77
Lymphocyte%	9.1	18.5
C-reactive protein(mg/dl)	158	61
Erythrocyte sedimentation rate(mm/hr)	120	53
Liver Function Tests		
Total bilirubin(mg/dl)	0.36	0.34
Direct Bilirubin(mg/dl)	0.15	0.19
Aspartate transaminase(U/L)	26	27
Alanine transaminase(U/L)	19	17
Alkaline phosphatase(U/L)	147	135
Gamma-glutamyl transferase(U/L)	16	18
Coagulation Profile		
Prothrombin time(seconds)	32	16
International normalized ratio	2.6	1.3
Activated partial thromboplastin time(seconds)	46	28
D-dimers(ng/dl)	756	243
Fibrinogen(g/L)	1.4	2.2
Basic Metabolic Panel		
Sodium(mEq/L)	138	136
Potassium(mEq/L)	3.9	4.2
Chloride(mEq/L)	99	96
Bicarbonate(mEq/L)	16	23
Creatinine(mg/dl)	0.7	0.8
Blood Urea Nitrogen(mg/dl)	8	9
Lactate(mmol/L)	5.6	2.4
Glucose(mg/dl)	114	119
Autoimmune profile	Negative	
Anti-nuclear antibodies		
Anti-double-stranded DNA		
Anti-Sjogren-syndrome A/B		
Anti-U1-Ribonucleic protein		
Anti-Scl-70		
Anti-Smith		

The patient was immediately shifted to the intensive care unit (ICU) and resuscitated with crystalloids and vasopressors given her shock. The patient was managed with analgesics and intravenous broad-spectrum antibiotics (Meropenem and Clindamycin) while awaiting cultures. Aspirin and low molecular weight heparin (LMWH) were commenced with close monitoring for bleeding. Plastic surgery and orthopaedic departments were taken on board.

The blood cultures revealed Staphylococcus aureus and Pseudomonas aeruginosa, while urine culture was normal. By the fifth day, her swelling and discoloration improved with sufficient wound healing. She underwent amputation for the gangrenous toes and fingers. The general condition of the patient and laboratory parameters improved. The patient was discharged upon completion of antibiotics with the resolution of symptoms. The patient was diagnosed with SPG secondary to postpartum sepsis and DIC.

## DISCUSSION

SPG is an infrequent complication of sepsis and DIC. It can develop due to postpartum sepsis. In developing countries, there is a major burden of postpartum sepsis due to unsafe birth practices carried out by unskilled midwives, and contaminated instruments in an unsterile environment.^4^ Common organisms yielded from blood cultures are Staphylococcus aureus, Klebsiella, Pseudomonas aeruginosa, Streptococcal pyrogens, and coagulase-negative Staphylococci.^5^ Staphylococcus aureus and Pseudomonas aeruginosa were also found in the culture of our patient. It is seen that postpartum sepsis is a recognized cause of DIC.^6^ Our patient developed postpartum sepsis progressing to DIC and symmetrical gangrene of all four limbs. The hypercoagulability during the postpartum period also contributed to DIC. It was clinically evident by acro-cyanosis and gangrene.

Tamboli S et al. highlighted a similar case of postpartum septic shock followed by peripheral gangrene.^5^ Another report by Miguel N. Albano et al. presents a case of SPG secondary to septic shock due to a urinary tract infection.^7^ The patient was managed similarly, followed by amputation of distal phalanges of fingers and toes. Hector Masaragian et al, present a case of four-limb gangrene resulting from septic shock after surgical rhinoplasty.^8^

SPG is a rare entity hence, no specific guidelines have been formulated for its management. The management is supportive with antibiotics, hydration, correction of electrolytes and coagulation profile, and treating the underlying precipitating factor.^9^ It is seen that early diagnosis can herald the progression of gangrene and limit morbidity, improving the quality of life.^9^ Anticoagulation plays a role in decreasing the pro-thrombotic state and prevents further deterioration. It is also important to maintain good cardiac output to improve peripheral limb circulation. Regrettably, established gangrene cannot be reversed despite good maintenance of blood flow which ultimately results in amputation.^7^,^8^ However, aggressive and prompt management limits progression to gangrene and reverses ischemia.

## CONCLUSION

Postpartum sepsis due to unskilled labor, unsafe birth practices, and unsterile instruments used during childbirth can progress to septic shock and DIC. This can be complicated by SPG. Therefore, it is important to be suspicious of SPG given any cyanosis or gangrene developing in the postpartum period. Early detection, prompt management, commencement of broad-spectrum antibiotics and use of low molecular heparin can prevent progression from pre-gangrenous to gangrenous stage and loss of limbs. It is also crucial to create awareness for prenatal and natal hospital care for early detection of high risk events.

### Authors’ Contribution:

**WB:** conceived the idea and drafting of case and responsible for integrity of the work.

**SN and SK:** drafting of introduction, discussion, and conclusion.

**SMA**: review and final approval of the article.

## References

[1] Sharma BD, Kabra SR, Gupta B (2004). Symmetrical Peripheral Gangrene. Trop Doctor.

[2] Foead AI, Mathialagan A, Varadarajan R, Larvin M (2018). Management of Symmetrical Peripheral Gangrene. Indian J Crit Care Med.

[3] Ghosh S, Bandyopadhyay D, Ghosh A (2010). Symmetrical peripheral gangrene:a prospective study of 14 consecutive cases in a tertiary-care hospital in eastern India. J Eur Acad Dermatol Venereol.

[4] Bakhtawar S, Sheikh S, Qureshi R, Hoodbhoy Z, Payne B, Iqbal A (2020). Risk factors for postpartum sepsis:a nested case-control study. BMC Pregnancy Childbirth.

[5] Tamboli S, Tamboli S, Shrikhande S (2017). Puerperal sepsis:predominant organisms and their antibiotic sensitivity pattern. Int J Reprod Contraception Obstet Gynecol.

[6] Molos MA, Hall JC (1985). Symmetrical Peripheral Gangrene and Disseminated Intravascular Coagulation. Arch Dermatol.

[7] Albano MN, Brazão SG, Caroço TV, Louro JM, Coelho MI, Almeida CEC (2018). Rare case of symmetrical peripheral gangrene due to septic shock, disseminated intravascular coagulation and inotropic use. Ann Med Surg (Lond).

[8] Masaragian H, Rega L, Ameriso N, Perin FD, Coria H, Mizdraji L (2019). Distal ischemia of the Four Limbs:A Case Report and Literature Review. J Foot Ankle Surg (Asia Pacific).

[9] Tripathy S, Rath B (2010). Symmetric peripheral gangrene:Catch it early!. J Emerg Trauma Shock.

